# Microbial dysbiosis and lack of SCFA production in a Spanish cohort of patients with multiple sclerosis

**DOI:** 10.3389/fimmu.2022.960761

**Published:** 2022-10-17

**Authors:** Laura Moles, Susana Delgado, Miriam Gorostidi-Aicua, Lucía Sepúlveda, Ainhoa Alberro, Leire Iparraguirre, Jose Alberto Suárez, Leire Romarate, Maialen Arruti, Maider Muñoz-Culla, Tamara Castillo-Triviño, David Otaegui, The international Multiple Sclerosis Microbiome Study Consortium

**Affiliations:** ^1^ Biodonostia Health Research Institute, Group of Multiple Sclerosis, San Sebastián, Spain; ^2^ Department of Microbiology and Biochemistry of Dairy Products, Instituto de Productos Lácteos de Asturias–Consejo Superior de Investigaciones Científicas (IPLA-CSIC), Villaviciosa, Spain; ^3^ Spanish Network on Multiple Sclerosis, Hospital Universitario Ramón y Cajal, Servicio de Inmunología, Madrid, Spain; ^4^ Neurology Department, Donostia University Hospital, Osakidetza Basque Health Service, San Sebastián, Spain

**Keywords:** multiple sclerosis, gut microbiota, short chain fatty acids, enterobacteria, butyrate

## Abstract

**Background:**

Multiple sclerosis (MS) is a chronic, demyelinating, and immune-mediated disease of the central nervous system caused by a combination of genetic, epigenetic, and environmental factors. The incidence of MS has increased in the past several decades, suggesting changes in the environmental risk factors. Much effort has been made in the description of the gut microbiota in MS; however, little is known about the dysbiosis on its function. The microbiota produces thousands of biologically active substances among which are notable the short-chain fatty acid (SCFA) excretion.

**Objectives:**

Analyze the interaction between microbiota, SCFAs, diet, and MS.

**Methods:**

16S, nutritional questionnaires, and SCFAS quantification have been recovered from MS patients and controls.

**Results:**

Our results revealed an increment in the phylum Proteobacteria, especially the family Enterobacteriaceae, a lack in total SCFA excretion, and an altered profile of SCFAs in a Spanish cohort of MS patients. These alterations are more evident in patients with higher disability.

**Conclusions:**

The abundance of Proteobacteria and acetate and the low excretion of total SCFAs, especially butyrate, are common characteristics of MS patients, and besides, both are associated with a worse prognosis of the disease.

## Background

Multiple sclerosis (MS) is a chronic, demyelinating, and immune mediated disease of the central nervous system (CNS) caused by a complex combination of genetic, epigenetic, and environmental factors. The incidence of MS has increased in the past decades, suggesting changes in the environmental risk factors. Among them, gut microbiota has received much attention, with several works describing a slight increase in *Firmicutes* and a reduction in Bacteroidetes phyla; an increase in members of the genera *Methanobrevibacterium*, *Akkermansia*, *Acinetobacter*, *Pseudomonas*, *Blautia*, and *Ruminococcus*; and a decrease in *Sutterella*, *Faecalibacterium*, *Prevotella*, *Fusobacterium*, *Anaerostipes*, *Clostridium* cluster XIVa and IV, *Parabacteroides*, and *Butyricimonas* genera (1, 2). Nevertheless, the intervariability of the microbiota and the heterogeneity of the disease make difficult the standardization of the microbial dysbiosis. Even more, diet, lifestyle, geographical location, and genetic background are known agents influencing the microbiota composition and could be confusing factors. To elucidate the alteration of the gut microbiota in MS, the international MS Microbiome Study (iMSMS) has recently validated the household paired recruitment to overcome common inherent limitations in microbiome research (3).

The functional role of the microbiota is based on the production of thousands of biologically active substances that can access the systemic circulation and reach their target tissues. Of special interest are the short-chain fatty acids (SCFAs) which are the main mediators of the interaction with the immune and neuroendocrine systems. The most abundant (95% of the total) produced SCFAs are acetate, propionate, and butyrate. Many of the microorganisms that produce these compounds are altered in MS patients; however, the study of their production in patients has been so far scarcely explored. SCFAs perform multiple functions in physiological, metabolic, and regulatory processes, and their adequate production is related to intestinal health and with the formation of an anti-inflammatory environment (4) (5). The participation of SCFAs in the homeostasis of lipid metabolism has also been described, whose loss can have consequences on the integrity of myelin, maturation of microglia, modulation of neuroprotection, and repair and remyelination of the CNS (6) (7). Besides, SCFAs have a prominent role in the regulation of the gut–brain axis, for their ability to modulate the neuroendocrine system and the production of several neurotransmitters (8) (9).

## Objectives

In this context, the aim of the current work is to characterize the particularities of the microbiota composition and SCFA production of an MS Spanish cohort and integrate them with their dietary habits.

## Methods

### Participants and sampling

Twenty patients with MS and 20 healthy controls that cohabit with them and are not genetically related have been recruited from the Hospital Universitario Donostia. Paired patients and control share (mostly) not only their diet but also the home environment. Written informed consent was obtained for each participant before inclusion. To be eligible for enrolment, participants must not suffer gastrointestinal or chronic infectious diseases, must not be pregnant or in a period shorter than 6 months after delivery, and must not have received steroids in the last month or chemotherapy or antibiotics in the last 3 months. Besides, patients must have a definite diagnosis of RRMS and must have been on the same disease-modifying treatment for the last 3 months. In the case of controls, they must not suffer any autoimmune disease. Demographic and clinical data are described in **Table 1**. All participants were provided with a dietary survey, which consist of a food-frequency questionnaire (developed and validated by NutritionQuest), and a kit for the collection of the feces at home. This kit contains a toilet stool catcher, two feces containers, a safety bag, a hydrated cold accumulator, and an isothermal bag for the transport. Participants were instructed about stool collection and transport. Fecal samples were immediately frozen at -20°C and were subsequently transported, together with the completed nutritional questionnaire, to the hospital protected with a cold accumulator. Once in the center, samples were stored at -80°C. A neurologist specializing in MS evaluated the patients and determined their disability according to the Expanded Disability Status Scale (EDSS), a method of quantifying disability and monitoring changes in the level of disability over time in MS.

**Table 1:** Clinical and demographic data of the studied population.

		Patient	Control
**Sex**	**Male**	4 (20%)	17 (85%)
	**Female**	16 (80%)	3 (15%)
**Age**		47.1 (47.0-47.2)	49.2 (49.1-49.3)
**BMI**	**Underweight**	0 (0%)	1 (5%)
	**Normal**	14 (70%)	9 (45%)
	**Overweight**	6 (30%)	10 (50%)
**Disease modifying treatment**	**0**	4 (20%)	–
	**IFN-β**	3 (15%)	–
	**Gilenya**	4 (20%)	–
	**Tecfidera**	9 (45%)	–
**Years of MS evolution**		15.0 (14.9-15,1)	–
**EDSS score**		1.9 (0-6.5)	–

INF-β, beta-interferons.

EDSS, Expanded Disability Status Scale.

BMI, body mass index.

### Bacterial DNA extraction and fecal microbiome study

Fecal samples were thawed on ice and diluted in 1× DPBS (Gibco, USA). DNA extraction was performed using mechanical and enzymatic lysis in combination with the kit QIAamp DNA Stool Mini Kit (Qiagen, Germany). Mechanical disruption was performed on the pelleted microbial cells by a three times treatment of bead-beating with 0.1-mm-diameter zirconia/silica beads (Sigma, St. Louis, USA) using a Tissue Lyser (Qiagen, Germany). Enzymatic lysis was done with lysozyme (Sigma, St. Louis, USA, 10 mg/ml). Negative control was performed in an empty sterile tube without adding any biological sample to evaluate the influence of reactants and possible contamination background.

The fecal microbiome study was performed on Ion Torrent equipment (Life Technologies, MA, USA). Between 300 and 400 ng of DNA was used to perform the amplification and sequencing. The Ion 16S Metagenomics Kit (Thermo Fisher, Waltham, MA, USA) was used to amplify 16S rRNA V2, V3, V4, V6, V7, V8, and V9 hypervariable regions. Barcode libraries were created using the Ion Plus Fragment Library Kit (Thermo Fisher, Waltham, MA, USA). The final library concentration was quantified (Kapa Library Quantification Kit (Roche, Basel, Switzerland)), and 26 pM of DNA was loaded onto Ion Torrent 318 chips.

### Quantification of fecal SCFAs

Acetic, butyric, propionic, isobutyric, valeric, isovaleric, and caproic acids were measured from the fecal waters obtained from 1 g of feces. This material was acidified with 1 ml of 20% (v/v) formic acid (PanReac, Barcelona, Spain), extracted with methanol (PanReac, Barcelona, Spain) and supplemented with 1 ml of 2-ethyl-butyric acid (Sigma, St. Louis, USA), used as an internal standard, and frozen at -80°C (10). Separation and quantification of the SCFAs were carried out in the supernatants after centrifugation and filtration by gas chromatography on a PE 8600 chromatograph (Perkin Elmer, Wellesley, MA, USA) using an HP-FFAP column (Hewlett-Packard, Palo Alto, CA, USA). The separation was carried out in a temperature range of 100°C to 220°C, and the detection was performed by flame ionization.

### Statistical analysis

Obtained reads were analyzed using the metagenomic workflow of the ion reporter software to associate them with bacterial genomes. The Shannon diversity index (SDI) was calculated by using the R vegan package. Sample clustering and statistical analyses were carried out in the R environment. Paired-sample Wilcoxon and paired-sample T tests were used to compare the microbial taxon abundance, SCFA profiles, and alpha diversity between the groups. Fisher’s exact test and the Freeman–Halton extension of the Fisher exact probability test were used to compare proportions. Principal component analysis (PCA) and PERMANOVA test were carried out in order to explore the relationship between the family-/genus-level and SCFA production datasets. Concurrently, the online resource Galaxy Huttenhower platform was used to determine the LDA Effect Size (LEfse algorithm) and get the cladograms in which are represented the microbial taxons that are significantly different in the samples.

## Results

### Characteristics of the participants and dietary habits

**Figure 1:**
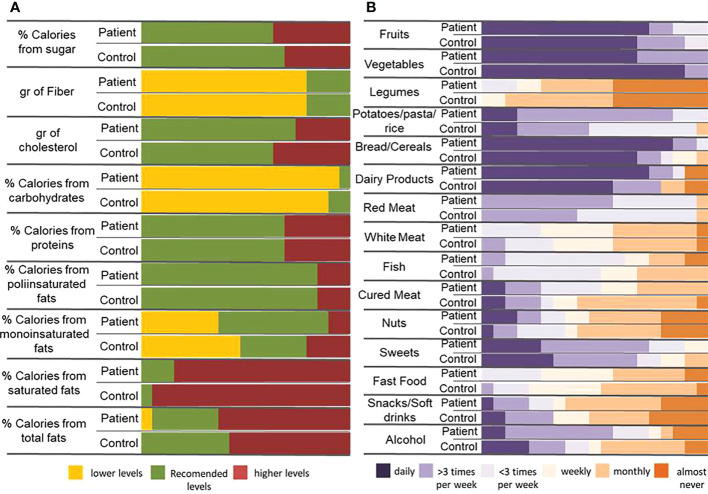
Dietary habits in patients and controls. **(A)** Percentage of calories provided by the main nutrients and contribution of some important components of the diet (cholesterol and fiber) in patients and controls. The reference data used to carry out the analysis has been 25-35% of de total calories provided by fats (being saturate fats less than 7%, monounsaturated fats 15-20% and polyunsaturated fats no more than 9%), 10-20% by proteins and 50-60% by carbohydrates. Sugars must not provide more than 10% of total calories and the consumption of less than 200g of cholesterol and between 20 and 35g of fiber are recommended. **(B)** Frequency of food intake in patient and control individuals.

Eighty percent of the patients were under treatment (**Table 1**). The household paired recruitment makes that most of the controls were the patients’ sentimental partner; consequently, our cohort showed an important gender imbalance, which has been considered as a confusing factor in subsequent analyses. Nutritional questionnaires evidenced slight differences between patients’ and controls’ diet, which in no case reached statistical significance. In general, participants consume an excess of fats (especially saturated ones) and proteins and an insufficient intake of carbohydrates and fiber (**Figure 1A**). Most of the participants consume daily fruits, vegetables, bread/cereals, and dairy products, with slight differences between patients and controls, but also showed high intake of red meat (50% with more than three times/week). The intake of products that must be occasional in a healthy diet as fast food, alcohol, sweets, cured meat, snacks, and soft drinks is also excessive (**Figure 1B**).

### Microbiota composition

**Figure 2:**
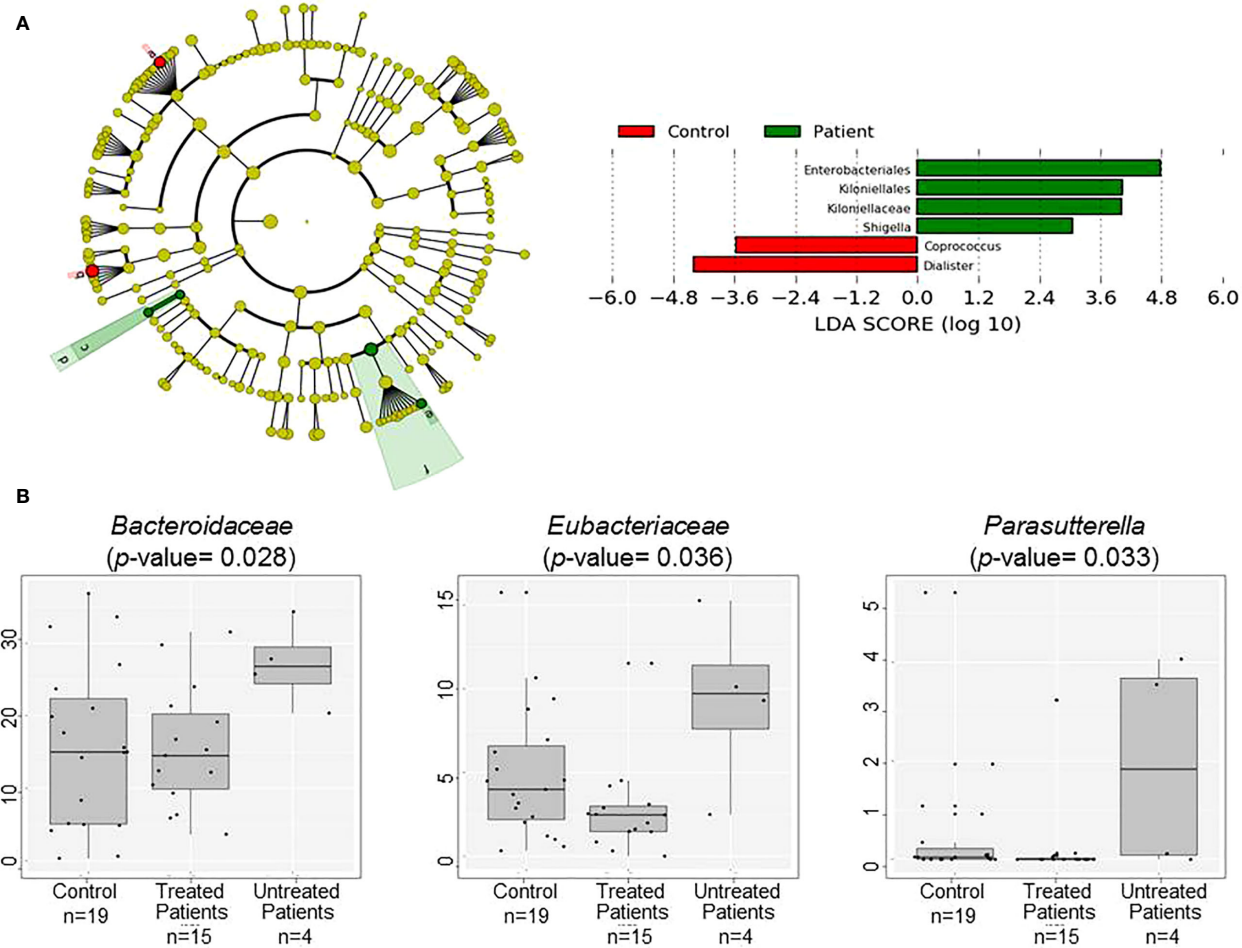
Comparison of patients’ and controls’ microbiota. **(A)** Cladogram plotted of the studied population’ microbiota. Cladograms show the different taxonomic levels by rings; the root of the cladogram denotes the domain bacteria, phyla were represented in the inner ring and genus in the outer one. The plotted represents the microbial differences in patients (green), and controls (red). On the right Linear discriminant analysis (LDA) analysis shows differentially abundant bacterial groups as biomarkers determined using Kruskal-Wallis test (P < 0.05) with LDA score > 2.0. a: Coprococcus, b: Dialister, c: Kiloniellaceae, d: Kiloniellales, e: Shigella, f: Enterobacteriales **(B)** Box plot showing the abundance of the main bacterial groups that differ in fecal samples of the patients grouped according to the used or not of disease modifying treatment and controls.

Results at the phylum level are shown in **Table 2**. No statistical differences between patients’ and controls’ microbiota could be observed at this taxonomical level; however, the tendency for the Proteobacteria phylum to increase in patients is remarkable. The detailed study of this bacterial group revealed the major presence and concentration of the family *Enterobacteriaceae* in patients (p-value 0.048). Regarding minor taxonomical levels, the decrease in the genera *Dialister* and *Coprococcus* (p-value 0.014 and 0.001 respectively) is noteworthy in patients (**Figure 2A**).

**Table 2:** Microbial phyla and α-diversity (Shannon diversity index) in the fecal microbiota of patients and controls in this study.

Microorganism	Treated patient		Untreated patient		Control		p-value^a^	p-value^b^
	n	Median (IQR)	n	Median (IQR)	n	Median (IQR)		
Actinobacteria	14 (93%)	0.75 (0.28; 2,19)	4 (100%)	1.00 (0.14; 1.91)	19 (100%)	1.09 (0.49; 2.51)	0.323	0.417
Bacteroidetes	15 (100)	31.34 (21.23-41.45)*	4 (100%)	35.49 (35.28- 35.70)*	19 (100%)	34.03 (33.87- 34.19)*	0.473	0.806
Firmicutes	15 (100)	39.64 (22.13-57.16)*	4 (100%)	39.64 (39.38- 39.90)*	19 (100%)	43.74 (43.58- 43.90)*	0.412	0.494
Proteobacteria	15 (100)	28.81 (13.30; 36.30)	4 (100%)	23.41 (20.33; 26.70)	19 (100%)	17.93 (14.73; 19.39)	0.232	0.029
Tenericutes	10 (67%)	0.22 (0.00; 1.14)	1 (25%)	0.00 (0.00; 0.01)	13 (68%)	0.15 (0.00; 1.04)	0.902	0.066
Synergistetes	7 (47%)	0.00 (0.00; 0.21)	2 (50%)	0.08 (0.00; 0.19)	8 (42%)	0.00 (0.00; 0.02)	0.412	0.474
Verrucomicrobia	6 (40%)	0.00 (0.00; 0.16)	2 (50%)	0.02 (0.00; 0.08)	7 (37%)	0.00 (0.00; 0.03)	0.539	0.645
Others	3 (20%)	0.00 (0.00; 0.00)	1 (25%)	0.00 (0.00; 0.02)	2 (11%)	0.00 (0.00; 0.00)	0.353	0.368
Shannon Diversity Index	–	2.09 (2.00; 2.24)	–	2.12 (2.08; 2.16)	–	2.11 (2.06; 2.19)	0.986	0.808

a Kruskal–Wallis or ANOVA test of treated patients vs. controls.

b Kruskal–Wallis or ANOVA test of untreated patients vs. controls.

*Mean (95% CI).

n, number of samples where a specific microbial phyla was detected.

IQR, interquartile range.

Group Others consist of those phyla that represent less than 1% of the microbiota in all the samples, and include Chlorobi, Fusobacteria, Lentisphaerae, and Spirochaetes phyla.

The presence of families *Bacteroidaceae* and *Eubacteriaceae* and the genus *Parasutterella* was higher in untreated patients (**Figure 2B**), but no differences have been found when EDSS or time of evolution was analyzed. Gender influenced the presence of *Desulfovibrionaceae*, *Enterobacteriaceae*, and *Eubacteriaceae* families, age affected *Veillonellaceae* members, and BMI had effects at higher taxonomical levels affecting Actinobacteria (especially the *Bifidobacteriaceae* family), Firmicutes (especially *Prevotellaceae* and *Ruminococcaceae* families), and Proteobacteria phyla (especially *Enterobacteriaceae* family). Interestingly, opposite tendencies were observed in overweight patients and controls (**Figure S1**).

### SCFA production

**Figure 3:**
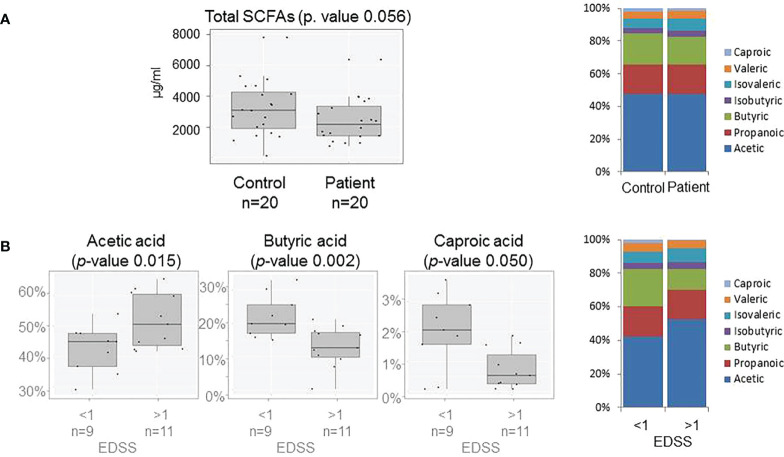
**(A)** Box plots showing abundance of total SCFAs in fecal samples (on the left) and SCFAs production (on the right) of patients and controls. **(B)** Box plots showing abundance of Acetic, Butyric and Caproic acids (on the left) and SCFAs profile (on the right) of patients grouped by EDSS score (>≤1).

The production of every measured SCFA and therefore the total excreted SCFAs in the controls was more abundant than in patients. The profile of SCFAs was similar in both groups; nevertheless, the production of butyric and caproic acids seems to be the most affected by (**Figure 3A**). Production of butyric acid (p-value 0.007) was affected by BMI and the excretion of isovaleric acid (p-value 0.047) by gender. Most of the produced SCFAs were less abundant in those patients with higher disability, with a decrease in the butyric and caproic acid production and the rise in acetic acid production while the EDSS score increased (**Figure 3B**).

### Influence of disease and diet on microbiota composition and SCFA production

**Figure 4:**
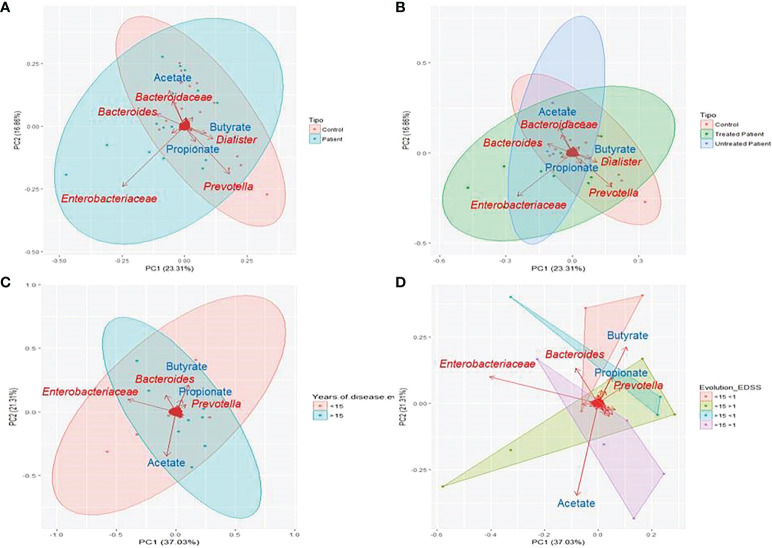
Principal component analysis (PCA) of microbiome results. PCA was performed based on the reads detected at each sample. Clustered samples were represented in different colours and the bacterial groups and produced SCFAs that had a higher influence placing the samples were represented with arrows. The bidimensional PCA plot **(A, B)** explains 40% of the sample’s variability. In **(A)** samples were clustered in patients (blue) and controls (red) (PERMANOVA P=0.218), while in **(B)** patients were divided on treated (green) and untreated (blue) (PERMANOVA P=0.199). PCA plot **(C, D)** explains 53.3% of the variability in the samples, that were clustered according to the years of disease evolution **(C)** in more or less than 15 years (blue and red respectively) and according to disease evolution (PERMANOVA P=0.675) **(D)** in less than 15 years of evolution and EDSS score more or less than 1 (green and red respectively) or more than 15 years of evolution and EDSS score more or less than 1(violet and blue respectively) (PERMANOVA P=0.38).

In the score plot containing the first and second PCs, which account for 40% of total variance, it was observed that the influence of the *Bacteroidaceae* family and *Dialister* and *Prevotella* genera and the production of acetate and propionate seem to direct the sample clustering of controls, while the family *Enterobacteriaceae* is the one with more influence in patients’ samples (**Figure 4A**). When patient treatment was considered, it was observed that treated and untreated patients formed two different clusters overlapped on PC1, where acetate production and the *Bacteriaceae* family have more influence on untreated patients (**Figure 4B**). The score plot representing the years of evolution shows that the first and second PCs account for 58% of the total variance. The first group (<15 years of evolution) seems to be directed by the presence of the *Prevotella* genus and the production of SCFAs (acetate, propionate, and butyrate), while the second group (>15 years of evolution) is mainly characterized by the presence of the *Enterobacteriaceae* family (**Figure 4C**). When the EDSS score values were also considered, it was noticed how the production of propionate and butyrate directed the cluster of patients with lower disability, while acetate production has major influence on those with higher disability (**Figure 4D**).

In order to evaluate how the main macronutrients of the diet influence on the composition and function of the microbiota, some further analyses were performed independently in patients and controls (**Table S1**). The percentage of calories provided by lipids in diet especially affects the presence of the Verrucomicrobia phylum (mainly represented by the genus *Akkermansia*) in patients. The concentration of valeric acid and the abundance of sequences belonging to the phylum Tenericutes were higher when the consumption of saturated fats is adjusted to the World Health Organization (WHO) dietary recommendations (<7%). The percentage of calories provided by the consumption of proteins especially affects the concentration of Bacteroidetes and Firmicutes phyla in patients. Carbohydrate intake seems to have minor influence on the microbiota composition and SCFA production (**Table S1**). **Figure S2** shows that the bacterial phylum that contributes more to total SCFA production was *Firmicutes* followed by *Actinobacteria* in both patients and controls.

## Discussion

In the last years, many studies have investigated the relationship between microbiota dysbiosis and MS. Most of them were carried out in the USA, where dietary habits are very different from those commonly found in Spain. The classical Spanish diet is Mediterranean diet, which has been considered beneficial for health maintenance and is characterized by high intake of cereals, legumes, nuts, vegetables, and fruits; fat consumption dominated by olive oil; moderate consumption of fish; low-to-moderate consumption of poultry and dairy products; low consumption of meat; and moderate alcohol intake, mainly red wine (11). Mediterranean diet promotes the presence of the genera *Prevotella*, *Roseburia*, *Ruminococcus*, and *Faecalibacterium*, microbial diversity, and a lower ratio Firmicutes/Bacteroidetes and shows numerous health beneficial effects (12). Nonetheless, eating habits in Mediterranean regions are already changing from the traditional patterns toward those of a Western pattern diet that involves a high intake of saturated fats and sucrose and a low intake of fiber, influencing the microbiota composition and driving diversity loss. Besides, the loss of species associated with anti-inflammatory conditions and the capacity to produce beneficial metabolites, including *Akkermansia muciniphila*, *Faecalibacterium prausnitzii*, *Roseburia* spp., *Eubacterium hallii*, *Clostridium* clusters XIVa and IV, and *Ruminococcus*, among others, have also been described (13, 14). Our data show that our population still maintains some of the healthy characteristics of the Mediterranean diet, as a diet based on the intake of fruits, cereals, and vegetables, but also revealed changes such as the poor consumption of fiber; the excessive intake of meat (especially red meat), sweets, and saturated fats; and the low consumption of legumes and nuts. This tendency could also be reflected on the increment of overweight and obesity in the Spanish population (15) that affects 40% of the participants.

It was previously described that the alteration in the microbiota composition of MS patients does not seem to affect the biodiversity parameters and is particularly tiny at the phylum level (1, 16). Moreover, it has been recently described that the ethnicity affects the microbiota composition (17) and the different treatments of the disease are able to modulate its composition in patients with MS (18). Our results showed similar tendencies in the case of diversity parameters and the bacterial groups *Blautia*, *Ruminococcus*, *Sutterella*, *Faecalibacterium*, *Prevotella*, *Fusarium*, *Anaerostipes*, and *Parabacteroides*, even if differences were not significant. The trends were contrary to those previously described in the case of *Clostridium* cluster XIVa and IV, *Butyricimonas*, *Akkermansia*, *Pseudomonas*, *Acinetobacter*, and *Methanobrevibacterium* (1, 2), but these bacterial groups were detected in a small number of samples and/or in insignificant concentrations. Besides, our results highlighted the increase in members of the Proteobacteria phylum, more specifically in the *Enterobacteriaceae* family in patients which, to our knowledge, has not been previously described in MS. This bacterial group includes several potential pathogens, and its expansion was related to a compromised ability to maintain a well-balanced microbiota, and therefore, it could serve as a potential diagnostic factor of bacterial dysbiosis (19). On the other hand, recent studies have associated a Proteobacteria expansion in the gut with high-fat diets and obesity (20). This tendency by itself cannot explain the different levels of Proteobacteria in both patient’s and control’s gut; however, the alteration of lipid metabolism in patients with MS has been recently published (6). It has been observed that the loss of homeostasis of lipid metabolism can have consequences on the integrity of myelin, the modulation of neuroprotection, and the repair and remyelination of the CNS. In fact, lipid metabolism has direct and indirect effects on the disability and progression of MS. Several studies show the association of high plasma levels of total cholesterol, triglycerides, and low-density lipoproteins (LDL) with greater disability. In addition, higher levels of products derived from lipid peroxidation in patients’ plasma have been described, suggesting the relationship of the disease with increased oxidative damage in lipoproteins (6). The association between lipid metabolism and specific bacterial groups are beginning to be reported. Schoeler et al. described the relation of elevated levels of lipopolysaccharide (LPS) in serum and higher levels of triglycerides, hepatic synthesis of VLDL, and reduction in lipoprotein catabolism (21). LPS is a structural component of the Gram-negative bacterial outer membrane. These bacteria are mainly represented in the gut by the phylum Proteobacteria. Besides, some groups of Proteobacteria, as the genus *Escherichia*, contribute to the bile acids and derivative oxidation (22). Considering these findings, the expansion of Proteobacteria in the patient’s gut could explain, at least in part, the alteration of the lipid metabolism in MS; nevertheless, these results must be validated in a larger population.

Besides, some differences were observed between patients and controls regarding dietary habits, showing that patients tend to follow a healthier diet which in addition seems to be associated with a lower rate of obesity. It is difficult to determine how these slight differences could affect the microbiota composition and functioning, but even if it is expected that major microbial changes are due to the disease itself, the influence of the particularities of each group diet must be considered. Our results evidence that the calories provided by fats in the diet could affect the concentrations of Verrucomicrobia and Tenericutes and the production of some SCFAs as valeric acid in patients but not in controls; even more, the tendencies observed in the control group were contrary to those described in patients. Similar results were observed considering the percentage of calories provided by proteins, which influence the presence of Bacteroidetes and Firmicutes on patients’ microbiota. These results seem to evidence that the metabolism alteration in patients with MS may be greater than previously thought.

At the lower taxonomical level, our results replicate some of the previously described tendencies in MS, such as the decrease of *Sutterella*, *Faecalibacterium*, *Prevotella*, *Anaerostipes*, and *Parabacteroides* and the increase of *Akkermansia* and *Blautia*. In addition, the reduction of *Dialister* and *Coprococcus* genera was also observed. Our data evidence the importance of factors as geographical location and diet in the microbiota studies. Therefore, larger studies are necessary to elucidate the real MS microbiota. In fact, the iMSMS was created with this objective and probably will shed some light on it in the following years (23).

To date, few studies evaluate the role of SCFAs in patients with MS. In our population, the analysis of the SCFA production revealed that both the total SCFA production and its profile are altered in MS. SCFAs are known to reduce inflammation and interact with the CNS participating in its regulation (7). Butyrate production seems to be the most affected, suffering the most dramatic depletion in patients with clinical acquired disability (EDSS >1). Previous studies pointed out the exacerbation of acetate in plasma of patients with MS and its correlation with the EDSS score (24). Besides, studies carried out in animal models have revealed the importance of butyrate in maintaining the integrity of the blood–brain barrier (25), regulating T-cell production and the expression of some miRNAs, such as miR-375, related with the disease progression (15). Even more, the treatment with butyrate in the cuprizone model facilitated oligodendrocyte maturation and enhanced remyelination (26), so it has a promising role on the MS course and as a potential target to be considered in future therapies. Regarding the influence of the dietary habits on SCFA production, our results showed a minor influence than expected in both patients and controls, pointing out the importance of the microbiota composition on its production. In this context, our results remark the association between a major presence of the phylum Bacteroidetes and family *Porphyromonadaceae*, and higher butyrate production in patients. The production of acetate in patients, which is associated with high EDSS, could also be related to a major presence of Firmicutes and the reduction in the family *Veillonellaceae*.

The results from this study allow relating the major presence of enterobacteria, the general loss of *Firmicutes* members, and the higher acetate and lower butyrate production not only with the MS but also with a worse EDSS. In this context, our work establishes a functional link between microbiota and MS and highlight the importance of a combined the study of diet, microbiome, and SCFA production.

## Data availability statement

The datasets presented in this study can be found in online repositories. The names of the repository/repositories and accession number(s) can be found in the article/**Supplementary Material**.

## Ethics statement

The studies involving human participants were reviewed and approved by Comité ético de investigación Hospital Universitario Donostia. The patients/participants provided their written informed consent to participate in this study.

## Author contributions

Conceptualization, LM, MM-C, TC-T, and DO; methodology, iMSMS (www.imsms.org), LM, SD, LS, LR, and MA; formal analysis, MG-A, LM, and SD; investigation, LM, SD, LI, AA, JS, MM-C, and DO; resources, SD and DO; writing—original draft preparation, LM, SD, MM-C, TC-T, and DO; writing—review and editing, all authors; funding acquisition, DO. All authors contributed to the article and approved the submitted version.

## Funding

This work was supported by the Spanish Network of Multiple Sclerosis (REEM) under the grant (BIOD19-021) and by Basque government projects (2018111038 and 2019111013).

## Acknowledgments

We would like to thank all the families that participated in this study.

## Conflict of interest

The authors declare that the research was conducted in the absence of any commercial or financial relationships that could be construed as a potential conflict of interest.

## Publisher’s note

All claims expressed in this article are solely those of the authors and do not necessarily represent those of their affiliated organizations, or those of the publisher, the editors and the reviewers. Any product that may be evaluated in this article, or claim that may be made by its manufacturer, is not guaranteed or endorsed by the publisher.

## Abbreviations

CNS, central nervous system; DPBS, Dulbecco’s phosphate buffered saline; EDSS, Expanded Disability Status Scale; iMSMS, International MS Microbiome Study; IFN-β, beta-interferons; IQR, interquartile range; LDA, linear discriminant analysis; MRI, magnetic resonance imaging; MS, multiple sclerosis; PCA, principal component analysis; RRMS, relapsing–remitting multiple sclerosis; SCFA, short-chain fatty acid.
