# Supplementary material for: Microbial dysbiosis and lack of SCFA production in a Spanish cohort of patients with multiple sclerosis
Source: Front Immunol. 2022 Oct 17;13:960761. doi: 10.3389/fimmu.2022.960761 (PMC9620961; doi:10.3389/fimmu.2022.960761)
Supplement: Supplementary file 1 [file DataSheet_1.docx]

| **Table S1. Influence of the diet in the microbiota composition and functioning (SCFAs production) in patients and controls.** | | | | | | |
| --- | --- | --- | --- | --- | --- | --- |
|  | Patients | |  | Controls | |  |
|  | Total lipids>35% | Total lipids<35% | p. value | Total lipids>35% | Total lipids<35% | p. value |
| n | 12 (67%) | 6 (33%) |  | 11 (61%) | 7 (39%) |  |
| Acetic | 48.52 (48.30-48.74) | 46.15 (46.03-46.27) | 0.652 | 48.12 (48.02-48.22) | 47.44 (47.33-47.55) | 0.789 |
| Propionic | 18.22 (18.11-18.33) | 16.73 (16.61-16.85) | 0.608 | 18.27 (18.21-18.33) | 17.94 (17.87-18.01) | 0.841 |
| Butyric | 17.19 (17.02-17.36) | 15.68 (15.57-15.79) | 0.718 | 17.57 (17.48-17.66) | 20.58 (20.53-20.63) | 0.141 |
| Isobutyric | 3.82 (3.79-3.85) | 5.09 (5.05-5.13) | 0.152 | 3.65 (3.62-3.68) | 3.03 (3.00-3.06) | 0.417 |
| Isovaleric | 6.80 (6.74-6.86) | 9.58 (9.48-9.68) | 0.144 | 6.23 (6.17-6.29) | 4.80 (4.75-4.85) | 0.324 |
| Valeric | 4.20 (4.18-4.22) | 5.74 (5.69-5.79) | 0.062 | 4.66 (4.65-4.67) | 4.00 (3.97-4.03) | 0.191 |
| Caproic | 1.26 (1.24-1.28) | 1.04 (1.01-1.07) | 0.706 | 1.52 (1.50-1.54) | 2.20 (2.16-2.24) | 0.367 |
| Total SCFAs | 2560.43 (2531.55-2589.31) | 2076.72 (2042.13-2111.31) | 0.534 | 2981.70 (2956.46-3006.94) | 3268.55 (3234.78-3302.32) | 0.671 |
| Actinobacteria | 0.46 (0.22; 1.88)* | 0.93 (0.43; 2.01)* | 0.303^ | 1.09 (0.60; 3.57)* | 1.07 (0.49; 2.29)* | 0.684^ |
| Bacteroidetes | 34.26 (34.09-34.43) | 27.67 (27.43-27.91) | 0.184 | 31.96 (31.73-32.21) | 38.32 (38.14-38.50) | 0.261 |
| Firmicutes | 37.10 (36.82-37.38) | 43.49 (43.04-43.93) | 0.446 | 43.91 (43.70-44.12) | 41.29 (41.04-41.54) | 0-629 |
| Proteobacteria | 24.30 (18.61; 35.14)* | 27.75 (16.40; 33.94)* | 0.925^ | 17.81 (16.70; 19.77)* | 19.19 (15.41; 19.39)* | 0.684^ |
| Tenericutes | 0.11 (0.00; 1.12)* | 0.41 (0.00; 0.96)* | 0.772^ | 0.37 (0.08; 1.33)* | 0.09 (0.00; 0.99)* | 0.461^ |
| Synergistetes | 0.00 (0.00; 0.18)* | 0.13 (0.02; 0.47)* | 0.317^ | 0.02 (0.00; 0.10)* | 0.00 (0.00; 0.01)* | 0.196^ |
| Verrucomicrobia | 0.00 (0.00; 0.01)* | 0.13 (0.02; 0.47)* | 0.043^ | 0.00 (0.00; 0.07)* | 0.00 (0.00; 0.01)* | 0.450^ |
| Others | 0.00 (0.00; 0.00)* | 0.04 (0.00; 0.21)* | 0.034^ | 0.00 (0.00; 0.00)* | 0.00 (0.00; 0.00)* | 0.246^ |
|  | Patients | |  | Controls | |  |
|  | Saturated fat>7% | Saturated fat<7% | p. value | Saturated fat>7% | Saturated fat<7% | p. value |
| n | 16 (89%) | 2 (11%) |  | 18 (100%) | 0 (0%) |  |
| Acetic | 48.09 (47.92-48.26) | 44.83 (42.84-46.82) | 0.767 | 47.86 (47.79-47.93) |  |  |
| Propionic | 18.26 (18.17-18.35) | 13.42 (12.82-14.02) | 0.473 | 18.14 (18.09-18.19) |  |  |
| Butyric | 16.99 (16.86-17.12) | 14.25 (13.62-14.88) | 0.736 | 18.74 (18.68-18.80) |  |  |
| Isobutyric | 3.98 (3.96-4.00) | 6.30 (6.02-6.58) | 0.132 | 3.41 (3.39-3.43) |  |  |
| Isovaleric | 7.09 (7.04-7.14) | 12.80 (12.23-13.35) | 0.073 | 5.67 (5.63-5.71) |  |  |
| Valeric | 4.41 (4.39-4.43) | 7.16 (6.84-7.48) | 0.022 | 4.40 (4.38-4.42) |  |  |
| Caproic | 1.18 (1.16-1.20) | 1.23 (1.18-1.28) | 0.528 | 1.78 (1.76-1.80) |  |  |
| Total SCFAs | 2550.64 (2526.82-2574.48) | 1187.64 (1134.98-1240.30) | 0.211 | 3093.26 (3073.51-3113.01) |  |  |
| Actinobacteria | 0.46 (0.22; 1.19)* | 3.96 (3.16; 4.76)* | 0.057^ | 1.08 (0.46; 2.70)* |  |  |
| Bacteroidetes | 32.62 (32.47-32.77)* | 27.67 (26.44-28.90) | 0.511 | 34.43 (34.26-34.60) |  |  |
| Firmicutes | 38.91 (38.64-39.18) | 41.77 (39.92-43.62) | 0.362 | 42.89 (42.73-43.05) |  |  |
| Proteobacteria | 27.14 (18.61; 35.14)* | 24.31 (18.71; 29.91)* | 0.570^ | 17.98 (16.60; 19.45)* |  |  |
| Tenericutes | 0.06 (0.00; 1.03)* | 1.48 (1.15; 1.81)* | 0.037^ | 0.26 (0.00; 1.09)* |  |  |
| Synergistetes | 0.00 (0.00; 0.19)* | 0.45 (0.26; 0.64)* | 0.233^ | 0.00 (0.00; 0.02)* |  |  |
| Verrucomicrobia | 0.00 (0.00; 0.07)* | 0.31 (0.20; 0.44)* | 0.191^ | 0.00 (0.00; 0.04)* |  |  |
| Others | 0.00 (0.00; 0.00)* | 0.04 (0.02; 0.06)* | 0.678^ | 0.00 (0.00; 0.00)* |  |  |
|  | Patients | |  | Controls | |  |
|  | Protein>20% | Protein<20% | p. value | Protein>20% | Protein<20% | p. value |
| n | 5 (28%) | 13 (72%) |  | 6 (33%) | 12 (67%) |  |
| Acetic | 44.18 (43.45-44.91) | 49.10 (48.93-49.27) | 0.369 | 47.87 (47.73-48.01) | 47.85 (47.76-47.94) | 0.992 |
| Propionic | 21.04 (20.81-21.27) | 16.45 (16.35-16.55) | 0.122 | 17.97 (17.87-18.07) | 18.23 (18.18-18.28) | 0.874 |
| Butyric | 18.70 (18.48-18.92) | 15.91 (15.76-16.06) | 0.522 | 18.90 (18.79-19.01) | 18.66 (18.59-18.73) | 0.910 |
| Isobutyric | 3.96 (3.90-4.02) | 4.35 (4.32-4.38) | 0.681 | 3.46 (3.42-3.50) | 3.38 (3.35-3.41) | 0.920 |
| Isovaleric | 6.73 (6.62-6.84) | 8.11 (8.04-8.18) | 0.503 | 5.66 (5.59-5.73) | 5.68 (5.62-5.74) | 0.991 |
| Valeric | 4.37 (4.30-4.44) | 4.84 (4.81-4.87) | 0.603 | 4.23 (4.19-4.27) | 4.49 (4.48-4.50) | 0.634 |
| Caproic | 1.03 (1.01-1.05) | 1.24 (1.22-1.26) | 0.729 | 1.90 (1.86-1.94) | 1.72 (1.70-1.74) | 0.818 |
| Total SCFAs | 2533.90 (2500.50-2567.30) | 2347.38 (2318.48-2376.28) | 0.821 | 3070.48 (3036.11-3104.85) | 3104.64 (3079.43-3129.85) | 0.961 |
| Actinobacteria | 0.27 (0.15; 0.75)* | 0.88 (0.29; 2.15)* | 0.183^ | 0.81 (0.33; 4.03)* | 1.39 (0.77; 2.32)* | 0.640^ |
| Bacteroidetes | 40.04 (39.63-40.45) | 29.00 (28.83-29.17) | 0.026 | 29.49 (29.18-29.80) | 36.91 (36.72-37.10) | 0.202 |
| Firmicutes | 27.03 (26.41-27.65) | 43.93 (43.65-44.21) | 0.043 | 43.69 (43.35-44.03) | 42.50 (43.32-42.68) | 0.832 |
| Proteobacteria | 34.48 (28.81; 37.09)* | 20.57 (13.48; 29.24)* | 0.183^ | 18.72 (14.06; 20.31)* | 17.92 (16.79; 19.21)* | 0.708^ |
| Tenericutes | 0.08 (0.00; 0.22)* | 0.14 (0.00; 1.10)* | 0.648^ | 0.50 (0.04; 1.06)* | 0.25 (0.00; 1.14)* | 1.000^ |
| Synergistetes | 0.00 (0.00; 0.00)* | 0.08 (0.00; 0.27)* | 0.155^ | 0.00 (0.00; 0.00)* | 0.01 (0.00; 0.06)* | 0.136^ |
| Verrucomicrobia | 0.00 (0.00; 0.00)* | 0.00 (0.00; 0.17)* | 0.287^ | 0.00 (0.00; 0.04)* | 0.00 (0.00; 0.02)* | 0.911^ |
| Others | 0.00 (0.00; 0.00)* | 0.00 (0.00; 0.07)* | 0.176^ | 0.00 (0.00; 0.00)* | 0.00 (0.00; 0.00)* | 0.304^ |
|  | Patients |  |  | Controls |  |  |
|  | Carbohydrates>50% | Carbohydrates<50% | p. value | Carbohydrates>50% | Carbohydrates<50% | p. value |
| n | 1 (6%) | 17 (94%) |  | 2 (11%) | 16 (89%) |  |
| Acetic | 42.25 | 48.05 (47.89-18.21) | 0.591 | 47.78 (47.69-47.87) | 47.87 (47.79-47.94) | 0.983 |
| Propionic | 14.46 | 17.91 (17.82-18.00) | 0.564 | 19.35 (19.31-19.39) | 17.99 (17.94-18.04) | 0.584 |
| Butyric | 17.65 | 16.63 (16.51-16.75) | 0.905 | 22.22 (22.21-22.23) | 18.30 (18.23-18.37) | 0.223 |
| Isobutyric | 5.98 | 4.14 (4.09-4.17) | 0.319 | 2.04 (2.04-2.04) | 3.58 (3.56-3.60) | 0.186 |
| Isovaleric | 11.69 | 7.49 (7.43-7.55) | 0.291 | 3.13 (3.12-3.14) | 5.99 (5.94-6.04) | 0.194 |
| Valeric | 7.43 | 4.55 (4.53-4.57) | 0.095 | 3.36 (3.34-3.38) | 4.53 (4.51-4.55) | 0.131 |
| Caproic | 0.54 | 1.22 (1.20-1.24) | 0.567 | 2.12 (1.99-2.25) | 1.74 (1.72-1.76) | 0.748 |
| Total SCFAs | 1582.38 | 2447.24 (2424.00-2470.48) | 0.590 | 3942.26 (3858.27-4026.25) | 2987.13 (2966.83-3007.43) | 0.356 |
| Actinobacteria | 0.88* | 0.50 (0.25; 2.03)* | 0.773^ | 1.06 (0.75; 1.37)* | 1.08 (0.49; 3.05)* | 0.673^ |
| Bacteroidetes | 32.84 | 32.02 (31.87-32.17) | 0.938 | 31.60 (31.09-32.11) | 34.79 (34.61-34.97) | 0.721 |
| Firmicutes | 24.69 | 40.09 (39.84-40.34) | 0.370 | 48.69 (48.26-49.12) | 42.17 (42.00-42.34) | 0.435 |
| Proteobacteria | 40.79* | 26.26 (15.55; 34.48)* | 0.210^ | 18.65 (18.34; 18.95)* | 17.87 (15.62; 19.78)* | 0.574^ |
| Tenericutes | 0 | 0.14 (0.00; 1.10)* | 0.275^ | 0.00 (0.00; 0.00)* | 0.56 (0.07; 1.20)* | 0.086^ |
| Synergistetes | 0.3 | 0.00 (0.00; 0.18)* | 0.181^ | 0.00 (0.00; 0.00)* | 0.01 (0.00; 0.04)* | 0.217^ |
| Verrucomicrobia | 0 | 0.00 (0.00; 0.17)* | 0.443^ | 0.01 (0.00; 0.01)* | 0.00 (0.00; 0.05)* | 0.933^ |
| Others | 0.5 | 0.00 (0.00; 0.00)* | 0.024^ | 0.00 (0.00; 0.00)* | 0.00 (0.00; 0.00)* | 0.607^ |
| *Median (IQR) | | | | | | |
| ^Kruskal-Wallis test | | | | | | |
