# Supplementary figures and images for: Microbial dysbiosis and lack of SCFA production in a Spanish cohort of patients with multiple sclerosis
Source: Front Immunol. 2022 Oct 17;13:960761. doi: 10.3389/fimmu.2022.960761 (PMC9620961; doi:10.3389/fimmu.2022.960761)

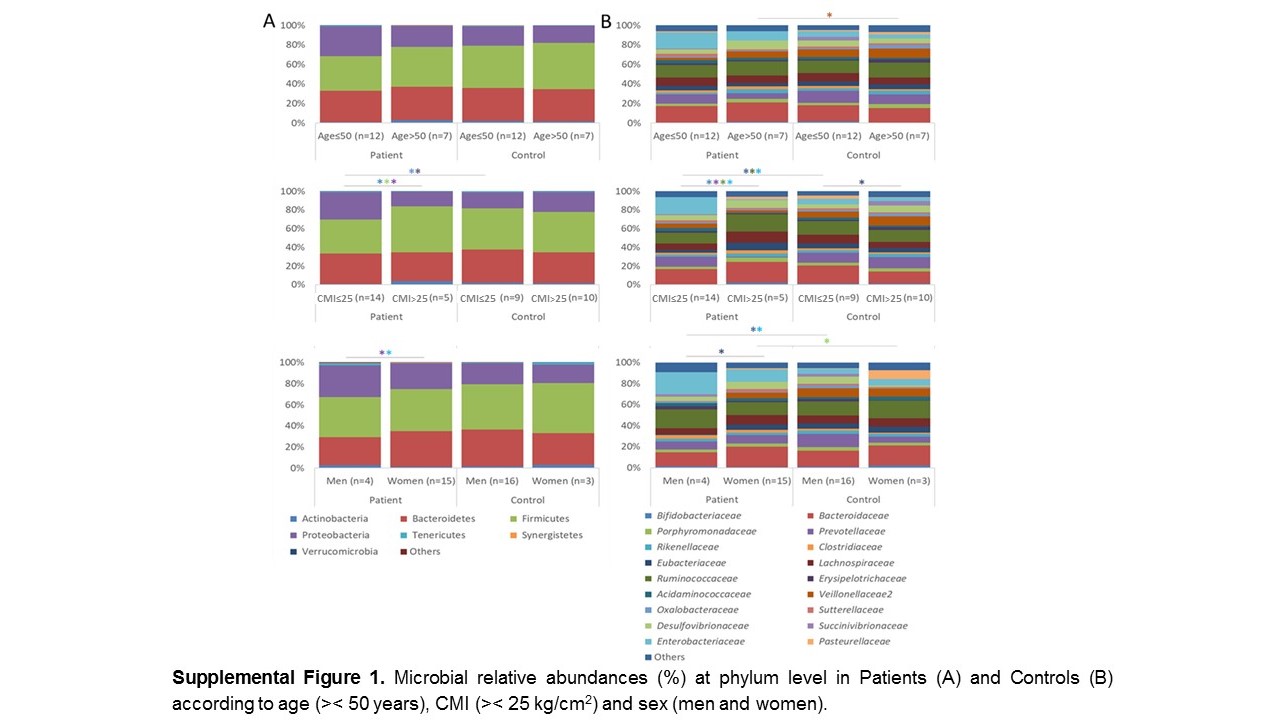

Supplement: Supplementary file 2 [file Image_1.jpg]

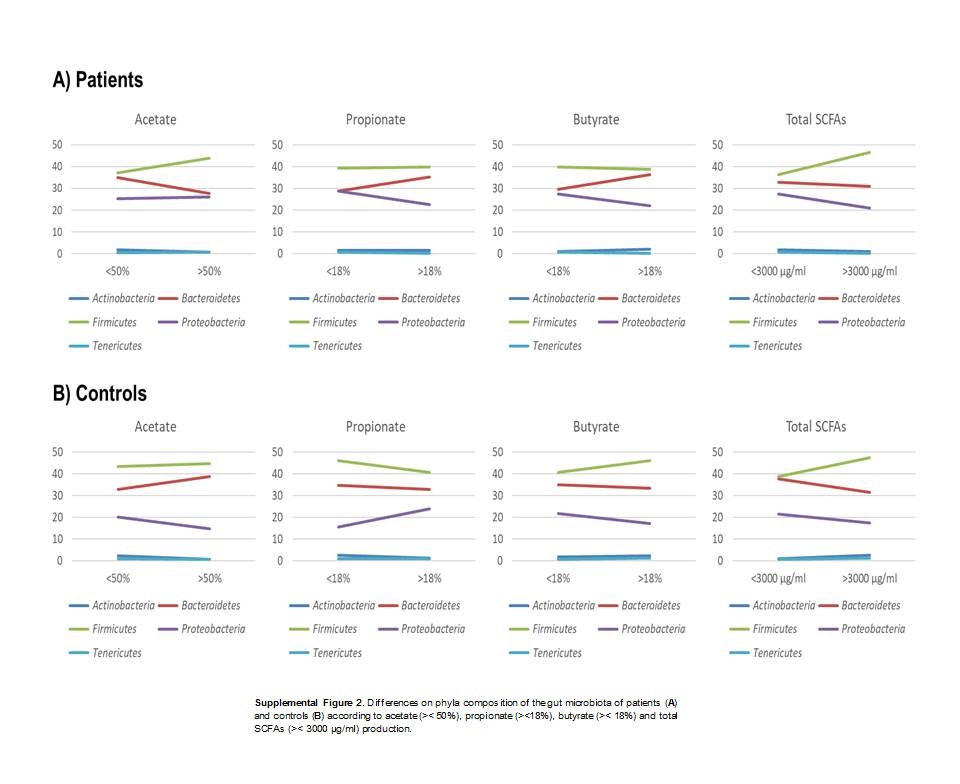

Supplement: Supplementary file 3 [file Image_2.jpeg]
